# Supplementary material for: Environmental Enrichment for Rainbow Trout Fingerlings: A Case Study Using Shelters in an Organic Trout Farm
Source: Animals (Basel). 2023 Jan 12;13(2):268. doi: 10.3390/ani13020268 (PMC9854733; doi:10.3390/ani13020268)

Supplementary Figure S1. Illustrative summary of the process of image analysis described in section 2.8.

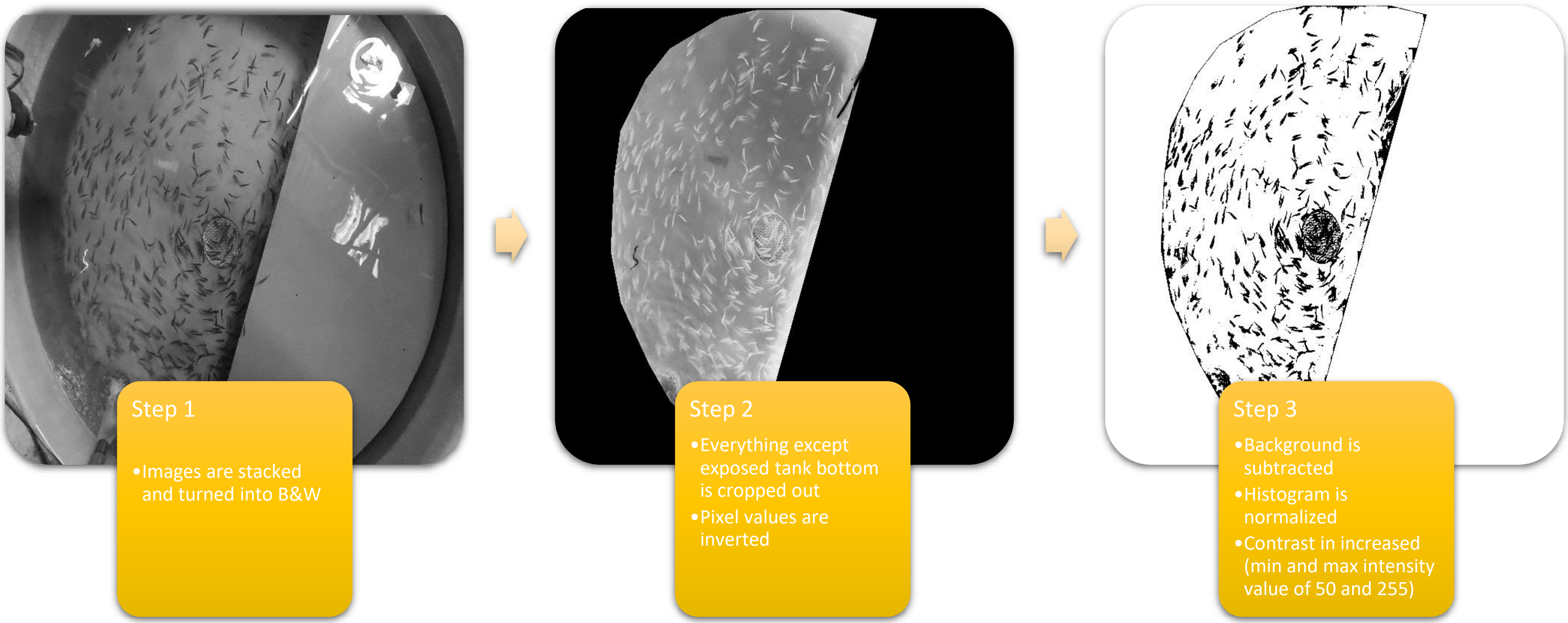

Supplement: Supplementary file 1 [file animals-13-00268-s001.zip › animals-2141289-supplementary.pdf]
